# High seroprevalence and high risk: why are older adults more prone to respiratory syncytial virus?

**DOI:** 10.1128/jvi.01432-25

**Published:** 2025-09-16

**Authors:** Piotr Rzymski, Barbara Poniedziałek, Dorota Zarębska-Michaluk, Krzysztof Tomasiewicz, Robert Flisiak

**Affiliations:** 1 Department of Environmental Medicine, Poznan University of Medical Sciences 37807, Poznań, Poland; 2 Department of Infectious Diseases and Allergology, Jan Kochanowski University 49693, Kielce, Poland; 3 Department of Infectious Diseases, Medical University of Lublin 49554, Lublin, Poland; 4 Department of Infectious Diseases and Hepatology, Medical University of Bialystok 37801, Bialystok, Poland; Indiana University Bloomington, Bloomington, Indiana, USA

**Keywords:** respiratory syncytial virus, immunosenescence, prefusion F protein, vaccination, older adults, antibody-dependent enhancement

## Abstract

Despite widespread seropositivity, respiratory syncytial virus (RSV) remains a major cause of severe illness in adults aged 60 years and older. This review examines why infection-acquired immunity fails to protect this group, focusing on four key factors: structural lung decline, comorbidities, immunosenescence, and impaired antibody responses. Age-related changes weaken mechanical defenses and antiviral immunity, while chronic diseases amplify RSV risk. Critically, repeated RSV infections may preferentially boost non-neutralizing antibodies targeting the postfusion F protein, limiting protection and possibly enhancing disease. The review also highlights how newly approved vaccines, based on stabilized prefusion F protein, can overcome these barriers by inducing strong neutralizing responses, offering a targeted strategy to reduce RSV burden in older adults.

## INTRODUCTION

Respiratory syncytial virus (RSV), classified in the family Pneumoviridae, is an enveloped, negative-sense, single-stranded RNA virus first isolated in 1956 from chimpanzees and, 1 year later, from children with bronchiolitis (1). Its characteristic ability to induce syncytium formation via the viral fusion (F) protein gave the pathogen its name. RSV circulates globally in predictable winter epidemics, infecting nearly all children by 2 years of age and reinfecting individuals throughout life. Antigenically, RSV is divided into two principal subgroups, A and B, defined by their G glycoprotein. Each consists of multiple, continuously evolving clades that co-circulate within a given season (2, 3). Although subgroup A strains generally predominate, both subgroups can circulate during outbreaks and contribute substantially to the overall disease burden, with current evidence showing no consistent differences in clinical severity between them (4–7).

RSV is among the leading causes of acute lower respiratory tract infection (LRTI) across the lifespan. Moreover, its reinfections are common and can occur within the same epidemic season (8–11). In 2019, the virus was estimated to cause more than 30 million LRTI episodes in children younger than 5 years worldwide, resulting in approximately 60,000 deaths (12, 13). A second, often under-recognized, burden peak is observed in adults aged 60 years and older. In the United States alone, RSV accounts for 123,000 to 193,000 hospitalizations and more than 10,000 deaths annually in this age group (14–17), with comparable age-specific morbidity documented in Europe (18) and Asia (19). The total annual costs of healthcare related to RSV in the United States were estimated to exceed $1 billion (20). Significantly, RSV disproportionatelyy affects individuals in low- to middle-income countries due to limited access to healthcare services, numerous clinical, socioeconomic, and environmental risk factors, and increased morbidity of HIV infection, which constitutes an additional severity risk (21–24).

High-risk groups for severe RSV disease include preterm infants, children with chronic lung or congenital heart disease, and immunocompromised individuals (25). Contemporary surveillance, however, identifies older adults, especially those with underlying health conditions, as an equally vulnerable population (26, 27). The disease burden in hospitalized older adults can exceed or be similar to that reported for nonpandemic influenza A, particularly among residents of long-term care facilities or individuals with chronic cardiopulmonary disease (28–30).

Although RSV was identified as a pathogen in 1957, progress toward an effective vaccine has been hindered by multiple setbacks (31, 32). The most notable failure occurred in the 1960s with a formalin-inactivated RSV vaccine, which unexpectedly caused enhanced respiratory disease in vaccinated children upon natural infection (33, 34). Approximately 80% of vaccine recipients required hospitalization, and two fatalities were reported. Subsequent studies demonstrated that formalin inactivation converted the F protein from its native prefusion to a postfusion conformation, exposing non-neutralizing epitopes (35). Therefore, the vaccine elicited antibodies with poor neutralizing capacity that contributed to antibody-dependent enhancement (ADE) of disease (36, 37). This experience delayed RSV vaccine research for decades.

Consequently, RSV management relied primarily on supportive care. Passive immunoprophylaxis with the monoclonal antibody palivizumab (approved in 1998) offered seasonal protection for a narrow subset of high-risk infants. It was impractical for widespread use because of its cost and monthly dosing schedule (38, 39). A significant advance came with the regulatory approval of nirsevimab in 2023 and clesrovimab in 2025 for infants and young children. These recombinant monoclonal antibodies target a conserved epitope on the F protein, have an extended serum half-life, and are administered intramuscularly as a single dose to protect for an entire RSV season (40–44).

For active immunization in adults, including older people, a pivotal breakthrough occurred in 2013 when stabilized prefusion structures were resolved, revealing neutralization-sensitive epitopes previously obscured in postfusion antigens that had dominated earlier vaccine efforts (45). Antigenic sites I–IV are present in both the pre- and postfusion F structures. In contrast, antigenic sites Ø and V (also known as site VIII) only occur in the prefusion conformation (45–47). Less than a decade after crystal structures of the F protein had been elucidated, two subunit vaccines based on recombinant prefusion F protein, AS01E-adjuvanted RSVPreF3 OA (Arexvy, GSK) and non-adjuvanted bivalent RSVpreF (Abrysvo, Pfizer), were authorized in 2023 for adults aged 60 years and older, each demonstrating greater than 80% efficacy against severe RSV-LRTI (48, 49). This was followed in 2024 by approval of an mRNA vaccine (mRNA-1345; mRESVIA, Moderna), encoding stabilized prefusion F protein, for older adults (50). A maternal formulation of Abrysvo has also been authorized to protect newborns via transplacental antibody transfer (51). Together with nirsevimab and clesrovimab, these products constitute the first comprehensive RSV prevention toolkit spanning infancy to late adulthood (39).

Serologic surveys, most of which were based on detecting IgG anti-RSV antibodies using enzyme-linked immunosorbent assay (ELISA), show that most adults older than 60 years possess RSV-specific antibodies, reflecting decades of repeated exposure (Table 1). Moreover, some studies demonstrate that their serum concentrations increase steadily with age (52). Nevertheless, incidence, hospitalization, and mortality continue to rise in this population (53–56). This narrative review explores why infection-induced adaptive immune responses are often insufficient to prevent severe RSV disease in older adults and evaluates how vaccination, particularly with prefusion-focused platforms, may reduce the substantial burden of the virus in this rapidly growing demographic.

**TABLE 1:** Seroprevalence of anti-RSV antibodies in different populations of elderly individuals^*a*^

Population	Age group (years)	*n*	Survey year	Assay	Seroprevalence	Reference
Poland	≥60	112	2023/24	ELISA	99% (IgG anti-RSV)	(52)
USA (Minnesota)	60–74	160	2022/23	ELISA	86% (IgG anti-F)	(57)
USA (Minnesota)	≥75	207	2022/23	ELISA	82% (IgG anti-F)	(57)
Korea	≥65	30	2021	ELISA	33% (IgG anti-preF)	(58)
India	61–70	44	2017/18	ELISA	93% (IgG anti-RSV)	(59)
India	71–85	55	2017/18	ELISA	98% (IgG-anti-RSV)	(59)
Italy	61–70	45	2008	IFA, MNA	89% (anti-RSV) 55% (neutralizing anti-RSV)	(60)
Italy	71–80	54	2008	IFA, MNA	91% (anti-RSV) 52% (neutralizing anti-RSV)	(60)
Italy	>80	58	2008	IFA, MNA	86% (anti-RSV) 36% (neutralizing anti-RSV)	(60)
Germany	60–69	52	Not reported	ELISA	96% (IgG anti-F)	(61)
Germany	70–79	19	Not reported	ELISA	95% (IgG anti-F)	(61)
Germany	80–89	6	Not reported	ELISA	83% (IgG anti-F)	(61)

^*a*^ IFA, indirect immunofluorescence assay; MNA, microneutralization assay; ELISA, enzyme-linked immunosorbent assay.

## WHY ARE OLDER PEOPLE MORE PRONE TO RSV INFECTION?

### Age-related changes in the respiratory system

With advancing age, the pulmonary system undergoes progressive anatomic and physiologic remodeling that diminishes respiratory reserve. Senile emphysema, characterized by loss of alveolar elastic recoil and airspace enlargement, lowers maximal expiratory flow and impairs oxygen diffusion, increasing vulnerability to lower respiratory tract infections (LRTIs), such as RSV (62, 63). These changes are compounded by calcification of thoracic joints and kyphotic curvature, which stiffen the chest wall and raise functional residual capacity (64). These structural alterations restrict the ability to compensate for ventilation-perfusion mismatch during acute viral infection (65, 66).

Aging also impairs mucociliary clearance, the first mechanical barrier to inhaled pathogens. Studies have demonstrated that ciliary beat frequency decreases significantly in older adults, and ultrastructural abnormalities become more common with age, reducing mucus transport efficacy (67, 68).

In parallel, expiratory muscle strength declines with age, impairing cough effectiveness. Age-related loss of airway surface liquid, attributable to epithelial dysfunction, further reduces the mobility of mucus (62, 69). This can impede the expulsion of RSV particles from the upper airway and synergize to allow deeper RSV penetration into the bronchioles and alveoli.

### Chronic diseases that amplify RSV risk

Aging is accompanied by a rising prevalence of chronic cardiopulmonary, vascular, metabolic, and oncologic disorders that synergistically increase RSV morbidity and mortality. These comorbidities impair host defenses, reduce physiologic reserve, and promote systemic inflammation, each contributing to a heightened risk of severe RSV disease in older adults.

Chronic obstructive pulmonary disease (COPD) and asthma are major risk factors for severe RSV infection. A recent systematic review estimated that their prevalence among RSV-infected adults in inpatient settings is 19% and 31%, respectively. Hospitalized COPD patients with RSV experience higher rates of respiratory failure and prolonged illness compared to non-infected counterparts (70–76). Moreover, the virus can exacerbate COPD by increasing airway inflammation, accelerating lung function decline, and contributing to disease progression (72, 75).

Congestive heart failure (CHF), ischemic heart disease, and a history of stroke also significantly raise the risk of severe RSV infection and related complications such as myocardial infarction (71, 72, 75–78). Data from RSV-NET (2015–2017) showed that adults ≥18 years with CHF had an adjusted hospitalization rate of 26.7 per 10,000 (compared with 3.3 per 10,000 in those without CHF), increasing to 40.5 per 10,000 in those ≥65 years, compared with 3.3 per 10,000 in those without CHF (79). RSV exacerbates cardiac stress by inducing hypoxia and systemic inflammation, worsening heart failure symptoms, and increasing the likelihood of ICU admission (80, 81). Importantly, the increased risk of cardiovascular events can persist for up to 6 months post-infection, especially for stroke and heart failure, though that risk gradually decreases over time (77, 82, 83).

Chronic kidney disease is associated with some of the highest RSV hospitalization rates among comorbidities (70, 78). Moreover, diabetes mellitus is frequently observed in elderly RSV patients and is associated with higher rates of severe disease and poor outcomes (70, 74). Both of these conditions can impair immune responses, increasing susceptibility to severe RSV-related complications, such as secondary bacterial pneumonia (81).

### Innate and adaptive immune senescence

With age, the human immune system undergoes a multidimensional functional decline known as immunosenescence, which impairs both antiviral defense and immunoregulation during RSV infection. This senescence affects the balance and activity of innate immune cells, reshapes adaptive T- and B-cell compartments, and fosters a state of low-grade chronic inflammation, often termed “inflammaging” (84–86).

Innate immune recognition is weakened by reduced expression of pattern recognition receptors (PRRs), such as Toll-like receptors (TLRs) and RIG-I-like receptors (RLRs), diminishing early detection of RSV and delaying the initiation of antiviral signaling cascades (87). Monocytes and macrophages in aged individuals exhibit reduced phagocytic activity and a shift toward pro-inflammatory phenotypes, producing elevated levels of IL-6, IL-1β, and TNF-α even in the absence of infection (87–89). This baseline hyperinflammation paradoxically coexists with impaired pathogen clearance and contributes to the immunopathology seen in older adults with RSV (90, 91).

Aging also impairs macrophage function, which in elderly individuals exhibits an altered response to stimuli, a declined ability to migrate, and remains in chronic activation, releasing increased levels of inflammatory cytokines, e.g., IL-1β and SASP cytokines (92, 93). At the same time, the expansion of CD16^+^ monocytes, which are more proinflammatory than classical monocytes, is observed in older adults (94). This is accompanied by the diminished production of antiviral type I interferons (93, 95). These changes can also contribute to increased susceptibility of the elderly to RSV infection and less effective viral clearance.

Importantly, T-cell immunity is also significantly compromised in the elderly (96). Thymic involution restricts the production of naïve CD4^+^ and CD8^+^ T cells, leading to a reliance on clonally expanded, often senescent memory populations with limited responsiveness to novel antigenic variants (97). The CD4^+^/CD8^+^ T cell ratio becomes skewed, and cytotoxic CD8^+^ T cells exhibit reduced proliferation and effector molecule secretion in response to RSV (98).

Regulatory T cells, whose frequency and suppressive activity increase with age (99), can also dampen effector responses to RSV antigens, potentially limiting viral clearance but exacerbating immunosuppression (100). This shift contributes to inadequate resolution of infection and prolonged viral shedding, which is known to be a marker of disease severity (101, 102). Moreover, the elderly show a significantly lower number of RSV F protein-specific IFN-γ-producing T cells and a reduced frequency of activated CD8^+^ T cells. Additionally, they exhibit higher IL-13 levels, which may indicate a skewed or less effective immune response (103). These findings suggest that impaired T-cell-mediated immunity also contributes to increased vulnerability to severe RSV disease in older adults.

Aging leads to a decline in naïve B cell output and reduced somatic hypermutation (97, 99), potentially impairing antibody diversity and affinity maturation against RSV. Memory B cell function is also compromised, resulting in shorter-lived and less effective humoral responses (104, 105).

Together, these changes culminate in a paradoxical immune landscape: increased baseline inflammation, defective early pathogen sensing, diminished cytotoxicity and release of antiviral molecules, and poor generation of protective antibody responses. All of these contribute to higher RSV susceptibility, prolonged disease, and poorer outcomes in older adults. 

### Antibody quality, antigenic bias, and the postfusion trap

Although seropositivity for RSV is nearly universal among adults aged 60 years and older (Table 1), it is now well established that antibody quality, not quantity, is the critical determinant of protection. Laboratory adsorption experiments using stabilized prefusion F vs postfusion F proteins have shown that only the former can elicit high neutralizing activity in human sera (106). It unequivocally emphasizes that the most potent neutralizing antibodies target prefusion F-specific epitopes.

However, the gradual loss of neutralizing activity of anti-RSV antibodies was shown to occur with age despite an increase in seropositivity, measured by a higher percentage of individuals with detectable antibodies (60). Therefore, repeated RSV exposure may drive an imbalanced immune response, favoring recall of non-neutralizing postfusion F-specific B cells over *de novo* responses to prefusion F. This possibility requires further investigatio,n though it would result from immunological imprinting, whereby the immune system preferentially recalls memory B cells targeting the more stable, immunodominant postfusion conformation of F protein encountered during initial childhood infections. Such imprinting may limit the formation of new antibody responses directed against neutralization-sensitive prefusion-specific epitopes in later life. Importantly, both prefusion F- and postfusion F-directed antibodies may bind with high affinity, but antibodies targeting the former conformation are far more effective at neutralization because they block the F protein refolding process required for viral membrane fusion.

In addition, immunosenescence impairs germinal center function, thereby reducing the overall capacity of aged individuals to generate diverse class-switched antibody repertoires (107–109). This impacts all antigenic targets, but the consequences are particularly important for RSV: if prefusion F-specific antibodies are not effectively induced, the neutralization capacity is markedly compromised, because antibodies that target postfusion F, although they may reveal high affinity, generally recognize epitopes less relevant for blocking viral entry. Thus, the issue is not that prefusion F antibodies are preferentially diminished, but that their absence or insufficiency leaves the host relying on functionally weaker postfusion F responses. Antigen presentation dynamics also play a role, as the prefusion F protein is structurally unstable and often transitions into the postfusion form before being processed by antigen-presenting cells, leading to a biased display of less protective epitopes (110). Consequently, repeated RSV infections may reinforce this postfusion-biased memory through clonal expansion of B cells targeting accessible but non-neutralizing regions. This immunological skewing results in elevated IgG binding titers that are functionally weak and poorly neutralizing. Supporting this, a prospective study in frail elderly persons showed that individuals who later developed symptomatic RSV infection had significantly lower preseason serum IgG titers to RSV F protein and lower neutralizing antibody titers to both RSV-A and RSV-B compared with matched controls who remained uninfected (111).

Importantly, animal studies have mechanistically linked low-quality post-vaccination antibodies to ADE, which had been observed historically with the formalin-inactivated RSV vaccine (36, 37). Furthermore, an *in vitro* study demonstrated that RSV co-incubated with suboptimal concentrations of neutralizing antibodies led to ADE and increased lung viral loads (112). Possibly, the similar suboptimal levels that occur in the elderly contribute to disease severity following subsequent RSV reinfections.

These findings highlight a central paradox in RSV immunity among older adults: despite near-universal seropositivity and sometimes elevated RSV-specific IgG titers, protection against infection and severe disease remains limited. This may not be due to a lack of antibodies *per se*, but rather to a qualitative decline in their functional capacity, marked by a shift toward non-neutralizing, postfusion F-biased responses that may even potentiate disease through mechanisms like ADE, as well as one or more of the physiological changes in older people. Table 2 summarizes mechanisms that may contribute to the predominance of weakly neutralizing immune responses against RSV in older adults. As such, these age-associated alterations in B cell memory and antibody quality underscore the urgent need for immunization strategies that selectively boost prefusion F-specific responses. Targeting these highly neutralizing epitopes may offer a more effective path to protective immunity in the elderly, a population especially vulnerable to RSV-related morbidity and mortality.

**TABLE 2:** Proposed mechanisms that may contribute to the age-related bias toward weakly neutralizing antibody responses in RSV immunity, highlighting areas requiring further investigation

Mechanism	Explanation
Immunological imprinting	Early exposures bias memory B cell responses to postfusion F
Immunosenescence	Reduced germinal center activity limits the generation of new neutralizing antibodies
Antigen instability	Prefusion F is unstable and less frequently presented
Clonal memory dominance	Repeated infections boost less effective postfusion-specific clones
Epitope exposure	Postfusion F exposes more immunodominant but non-neutralizing regions

## THE BENEFIT OF RSV VACCINATION IN THE ELDERLY

**Fig 1:**
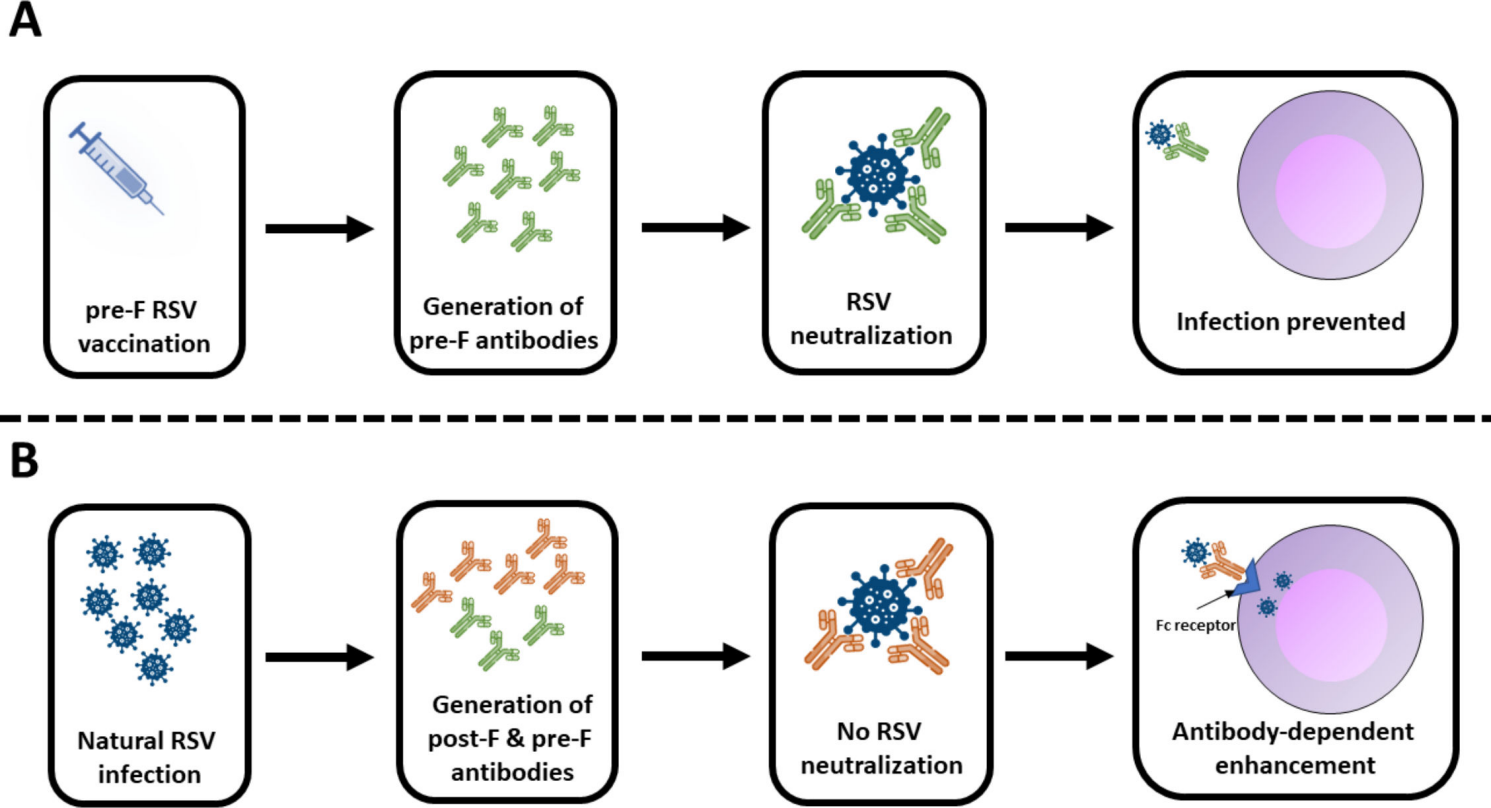
Difference in quality of humoral responses against the fusion (F) protein of RSV following vaccination (**A**) and natural infection (**B**) in older people. Vaccination leads to the generation of neutralizing anti-prefusion F antibodies (colored in green). At the same time, natural infection induces a broader response that includes neutralizing antibodies to prefusion F as well as cross-reactive antibodies with higher apparent affinity for postfusion F (colored in red). The latter contributes less effectively to viral neutralization and, in some contexts, may even facilitate antibody-dependent enhancement, which in elderly individuals could exacerbate disease severity due to comorbidities and immunosenescence.

All three vaccines currently available (Table 3) focus the immune responses on the prefusion F protein, which displays the most potent neutralizing epitopes (Table 3). Although natural RSV infection predominantly induces memory B cells specific for prefusion F, a substantial fraction of cross-reactive antibodies show higher apparent affinity for the postfusion form (113); these postfusion F-biased responses are generally less efficient at neutralization and, under certain conditions, may contribute to ADE (36, 37, 114) (Fig. 1). A seroprevalence survey that also focused on neutralization aspects clearly shows that despite nearly universal anti-RSV IgG seropositivity in older people, the serum of many of them may not exhibit any neutralizing activity against the virus (60). This leaves these individuals vulnerable not only to infection but also to its severe clinical course due to the accumulation of other factors, including underlying diseases and immunosenescence. In contrast, vaccines focusing on prefusion F protein as a target elicit a higher ratio of neutralizing to binding antibodies, effectively blocking viral fusion without enhancing disease severity (45, 115–117). Importantly, all authorized vaccines are based on a stabilized form of this protein, which is conformationally locked in the prefusion form and engineered to resist potential shifts to postfusion form and has preserved neutralizing epitopes (50, 118, 119). Importantly, the half-life of weakly neutralizing antibodies is shorter than that of antibodies with high potencies (120). Altogether, this explains the superiority of RSV vaccination in providing protection in the elderly. In addition, all authorized RSV vaccines also activate the cellular immune response, stimulating memory B cells and polyfunctional CD4^+^ T cells, with evidence that mRNA-1345 additionally stimulates specific CD8^+^ T cell responses (121–123). This cellular activation is important because it broadens the immune repertoire, supports more durable protection, and may enhance the response upon subsequent exposure to RSV.

**TABLE 3:** Characteristics of authorized RSV vaccines for older adults^*j*^

Code name	RSVPreF3 OA	RSVPreF	mRNA-1345
Commercial name	Arexvy	Abrysvo	mRESVIA
Manufacturer	GSK	Pfizer	Moderna
Approval	2023	2023	2024
Platform	Recombinant preF protein + AS01E adjuvant	Bivalent recombinant preF protein	mRNA encoding stabilized preF protein
Antigen	Prefusion F protein of RSV A2	Prefusion F proteins of RSV A2 and B (bivalent)	Prefusion F protein of RSV A2
Adjuvant	Yes (AS01E)	No	No
Schedule	Single dose	Single dose	Single dose
Elicited adaptive immune responses	Humoral (neutralizing antibodies); cellular (CD4^+^ T cells)^*a*^	Humoral (neutralizing antibodies); cellular (CD4^+^ T cells)^*b*^	Humoral (neutralizing antibodies); cellular (CD4^+^ and CD8^+^ T cells)^*c*^
Efficacy vs RSV-LRTD (first season)^*d*^	>80%^*e*^	>80%^*f*^	>80%^*g*^
Duration of protection	>1 season documented^*h*^	>1 season documented^*i*^	Ongoing study

^*a*^ (121).

^*b*^ (122).

^*c*^ (123, 124).

^*d*^ Direct head-to-head comparison is not possible due to different outcome definitions employed in the clinical trials; see reference 125.

^*e*^ (126).

^*f*^ (127).

^*g*^ (50).

^*h*^ (126).

^*i*^ (127).

^*j*^ RSV-LRTD, RSV-associated lower respiratory tract disease.

Notably, inter-individual variability in vaccine-induced immunity is substantial in older and immunocompromised adults, with some mounting robust neutralizing antibody responses, while others respond only weakly. A small exploratory study in immunocompromised individuals suggested that AS01E-adjuvanted formulation Arexvy may elicit higher neutralizing antibody titers than the non-adjuvanted vaccine (128). However, these preliminary findings are based on limited sample sizes, and any firm conclusions or preference for a specific product would require further investigations. It should also be noted that aging-related immunosenescence may diminish TLR signaling, but this is unlikely to compromise the action of the AS01E adjuvant. As a TLR4 agonist, it engages pathways that remain relatively well preserved in older adults, and by orchestrating broader, spatially coordinated immune activation in the lymph node, it compensates for age-related deficits (129–131).

Clinical trials have shown substantial efficacy across all three vaccines. In the phase 3 AReSVi-006 trial, vaccination of adults aged 60 years and older with Arexvy demonstrated 82.6% efficacy against RSV-associated lower respiratory tract disease (RSV-LRTD; defined as two or more lower respiratory symptoms/signs, including ≥1 lower respiratory sign, or three or more lower respiratory symptoms, lasting 24 h or longer) in the first RSV season and 56.1% in the second season, with a pooled efficacy of 74.5% over two seasons (126). Efficacy against medically attended LRTD was 87.5%. Efficacy over two seasons of a first dose followed by revaccination was 67.1% against RSV-LRTD and 78.8% against severe RSV-LRTD (126). The phase 3 RENOIR trial of Abrysvo, vaccination of individuals ≥60 years, resulted in 88.9% and 77.8% efficacy against RSV-LRTD with more than three symptoms over the first and second seasons, respectively. The efficacy against acute respiratory illness was 62.2% after the first season and 36.9% during the second season (127). In the case of mRESVIA, the efficacy against the disease with at least three symptoms in the ConquerRSV trial of adults aged ≥60 years was 82.4% during the first season, while the efficacy against acute respiratory disease was 68.4% (50). Direct head-to-head comparisons among these vaccines are not available, as the clinical trials used different outcome definitions and study designs (125); nonetheless, all authorized RSV vaccines have demonstrated substantial efficacy and should be considered effective, despite differences in technological platforms and composition.

Real-world observational studies further support these findings, with vaccine effectiveness (VE) estimates ranging from 75% to 80% against RSV-related acute respiratory infections, emergency department visits, and hospitalizations in older adults (132–134). Immunocompromised individuals aged over 60 years showed reduced but still clinically meaningful protection. VE against RSV-associated urgent care visits or hospitalizations was 67.0% for the group aged 60–74 years and 73.1% for those aged 75 years and older, with the lowest VE observed in the subgroup of patients who received stem cell transplants (133). Since immunocompromised individuals, including those with age-related immune deficiencies, may mount diminished responses to RSV vaccines while remaining at increased risk of severe disease, additional vaccination strategies in this group, such as revaccination or higher antigen formulations, could be considered in the future.

Both clinical trials and post-authorization surveillance indicated a generally favorable safety profile of RSV vaccines authorized for older people, with the most common side effects being mild to moderate, such as injection-site pain, fatigue, and headache (135). Some studies have reported a small but statistically significant increase in Guillain-Barré syndrome (GBS) cases (ranging from 5.2 to 18.2 per million doses in one study to 6.5–9.0 per million in another), although the overall risk remains low (133, 136). The potential mechanisms and contributing factors underlying this association are not yet fully understood and warrant further investigation. Importantly, and in line with expectations, the existing clinical and real-world studies indicate no evidence of ADE in adults vaccinated against RSV with prefusion-based formulations.

It is currently unknown how RSV vaccination may influence imprinting or the immune response to subsequent infections with circulating RSV strains. Concepts, such as original antigenic sin, in which the initial exposure shapes future immune responses, suggest that the age and prior infection history of the host could modulate vaccine-induced immunity, but this remains largely theoretical in the context of RSV (137).

In summary, RSV vaccination in the elderly provides robust protection against severe disease, with real-world data reinforcing clinical trial results. From a public health perspective, modeling studies suggest that RSV vaccination in older adults could reduce hospitalizations by 35%–64% in high-income countries, with substantial cost savings (138). Ongoing monitoring is necessary to assess long-term durability and rare adverse events, but current evidence strongly supports its use in this vulnerable population.

## CONCLUSIONS

Older adults are disproportionately vulnerable to severe RSV disease due to the combined effects of age-related lung degeneration, multimorbidity, immunosenescence, and a skewed antibody repertoire dominated by non-neutralizing responses. Together, these mechanisms explain why naturally acquired immunity, despite repeated exposures and high seroprevalence, often fails to confer protection in later life. Understanding these interacting factors underscores the clinical and immunologic value of prefusion F-based vaccination. Such vaccines elicit high-quality neutralizing antibodies and show strong efficacy in both clinical trials and real-world studies. To reduce global RSV-related morbidity and mortality in older populations, wider vaccine adoption, coupled with continued monitoring of safety and real-world effectiveness, is now a public health priority.
